# Manipulation of crossover frequency and distribution for plant breeding

**DOI:** 10.1007/s00122-018-3240-1

**Published:** 2018-11-27

**Authors:** A. Blary, E. Jenczewski

**Affiliations:** 1 Institut Jean-Pierre Bourgin, INRA, AgroParisTech, CNRS, Université Paris-Saclay, 78000 Versailles, France; 2 Department of Plant Breeding, IFZ Research Centre for Biosystems, Land Use and Nutrition, Justus Liebig University, Heinrich-Buff-Ring 26-32, 35392 Giessen, Germany

## Abstract

The crossovers (COs) that occur during meiotic recombination lead to genetic diversity upon which natural and artificial selection can act. The potential of tinkering with the mechanisms of meiotic recombination to increase the amount of genetic diversity accessible for breeders has been under the research spotlight for years. A wide variety of approaches have been proposed to increase CO frequency, alter CO distribution and induce COs between non-homologous chromosomal regions. For most of these approaches, translational biology will be crucial for demonstrating how these strategies can be of practical use in plant breeding. In this review, we describe how tinkering with meiotic recombination could benefit plant breeding and give concrete examples of how these strategies could be implemented into breeding programs.

## Introduction

Innovations in crop management practices and plant breeding have led to a steady increase in crop productivity over the years. Current progress in crop productivity is, however, not sufficient to cope with increasing food demand (Ray et al. 2012, 2013). Feeding a population of nine billion people by 2050 would require an additional 60–110% increase in crop production (Alexandratos and Bruinsma 2012). In the meantime, global warming is expected to reduce yields (Zhao et al. 2017). Plant breeding is one efficient way to achieve food security but this requires adoption of new technology and practices to boost crop production (Li et al. 2018) by capturing or generating more genetic diversity.

Conceptually, plant breeding aims to create new varieties that outperform the parents by combining their valuable traits. Crop improvement thus requires “*reshuffling of the genome to produce new favorable gene combinations in the progeny*” (Moose and Mumm 2008). Reshuffling of genetic information occurs during meiosis, i.e., the specialized cell division that leads to the production of gametes. During meiosis, formation of crossovers (COs), which are one of the products of meiotic recombination, leads to reciprocal exchanges of genetic information. CO formation in plants is a highly regulated process, which imposes a number of constraints for plant breeding. COs form at a low frequency, in preferential regions of the genome and are sensitive to sequence homology. Modifying CO patterning could thus be of great interest for breeders (Wijnker and de Jong 2008).

Increasing knowledge has been gained on the underlying molecular mechanisms that govern CO formation. In plants, studies in model species such as *Arabidopis thaliana* (thale cress) and to a lesser extent *Oryza sativa* (rice) and *Zea mays* (maize) have contributed to a more comprehensive view of meiotic recombination. This has led to the identification and functional analysis of more than 80 genes involved in meiosis (Mercier et al. 2015). This improved knowledge is beginning to make the promise of manipulating the mechanisms of meiotic recombination a reality for crop improvement. However, for this to happen the specific characteristics of different plant genomes, such as the abundance of repeated sequences or polyploidy, will need to be taken into account.

All flowering plants have a polyploid ancestry and up to 25–30% of extant flowering plants are extant polyploids (Alix et al. 2017; Jiao et al. 2011). Past and recent evidence support an adaptive significance of polyploidy in crop domestication and improvement (Hilu 1993; Salman-Minkov et al. 2016). Indeed, many important crops are very recent autopolyploids that arose from within-species whole-genome duplications (e.g., potato, alfalfa, some bananas) or allopolyploids, which have an interspecific hybrid origin (e.g., wheat, cotton, oilseed rape, coffee). One consequence of all these past and present polyploidy events is that all crop genomes are replete with duplicated genes and regions. These duplications, which range from short stretches of genes to complete chromosomes, impose additional constraints on correct chromosome segregation (due to multichromosomal interactions) and translational research into meiotic recombination (e.g., making it more difficult to identify functional homologues). Plant genomes also often contain a lot of transposable elements (Bennetzen and Kellogg 1997). In many species, transposable elements, usually retrotransposons, have accumulated in heterochromatic regions (Vitte et al. 2014) and contributed to the structural and functional partitioning of large chromosomes (Choulet et al. 2014). As proposed by Higgins et al. (2014), it is important to consider this global chromatin organization when it comes to understanding CO distribution in crops with large genomes (e.g., barley, tomato, wheat).

The aim of this review is to highlight the latest developments in translational biology for controlling CO formation in plant breeding programs. Firstly, we briefly introduce meiosis and meiotic recombination in plants. We then discuss how tinkering with CO patterning processes could benefit plant breeding. To this end, we provide an updated summary of the latest development in approaches aiming to increase CO frequencies between homologous and non-homologous chromosomes or alter CO position. We finally highlight the need for translational biology and discuss the relevance of these technologies in breeding programs.

## Control of CO formation in plants

Following the seminal works of Ross et al. (1997) and Klimyuk and Jones (1997), great strides have been made in elucidating the basic molecular mechanisms of meiosis and meiotic recombination in plants. As a series of recent reviews have provided comprehensive insights into these processes (Lambing et al. 2017; Lambing and Heckmann 2018; Lawrence et al. 2017; Mercier et al. 2015; Mézard et al. 2015; Ziolkowski and Henderson 2017), we will only briefly outline some key aspects here.

### The molecular mechanisms of meiotic recombination

Meiosis leads to the segregation of maternal and paternal (i.e., homologous) chromosomes through two successive cellular divisions that are preceded by a single round of DNA replication. This is achieved through bending the mitotic cell cycle rules to prevent an intervening replication between the two meiotic divisions (detailed in Mercier et al. 2015).

Proper chromosomal segregation during meiosis is highly dependent on two features: (1) precise control of sister chromatid cohesion and chromosome orientation and (2) establishment of physical connections between every pair of homologous chromosomes, which thereby form bivalents.

The first feature relies on a two-step release of sister chromatid cohesion associated with a change in kinetochore orientation. As soon as sister chromatids arise from meiotic DNA replication, they are held together by a ring-shaped protein complex that fosters cohesion (Bolaños-Villegas et al. 2017; Uhlmann and Nasmyth 1998). At the end of Meiosis I, sister chromatid cohesion is released from chromosome arms but remains protected at the centromere (Cromer et al. 2013; Zamariola et al. 2014). During Meiosis II, this protective effect is no longer assured, and sister chromatid cohesion is completely released. In the meantime, kinetochore orientation changes drastically between Meiosis I and Meiosis II. During Meiosis I, sister kinetochores attach to microtubules emanating from the same spindle pole while they attach to microtubules originating from the two different spindle poles during Meiosis II. These coordinated changes in sister chromatid cohesion and kinetochore orientation allow stepwise segregation of homologous chromosomes, then sister chromatids during Meiosis I and Meiosis II, respectively.

The establishment of “connections” between homologous chromosomes relies on meiotic recombination, the process in which programmed DNA double-strand breaks (DSBs) catalyzed by SPO11 proteins (Grelon et al. 2001; Robert et al. 2016) are repaired using intact sister or non-sister homologous chromatids as templates. Meiotic recombination results either in reciprocal exchanges of large DNA fragments (i.e., COs) or non-reciprocal exchanges of small patches of DNA between homologous chromatids with no exchange of flanking chromosome arms (i.e., NCOs) (Holliday 1964). COs are necessary for bivalent formation; thus, precise control ensures that at least one CO is formed between every pair of homologues during wild-type (WT) meiosis (“obligatory CO”). The universal phenomenon by which DSB repair is biased, at least to some extent, toward the homologous chromosome remains poorly understood (reviewed in Lambing et al. 2017; Mercier et al. 2015). Despite this bias toward the homologous chromosome, only a few DSBs are resolved as COs during WT meiosis; the vast majority of DSBs are repaired either as non-COs or by sister chromatids (De Muyt et al. 2009). In plants, as in animals and budding yeast, all the early DSB-initiated inter-homologue interactions, including those that are resolved as non-COs, are thought to be instrumental for promoting homologue recognition (Zickler and Kleckner 2015). This process of meiotic partner recognition is more intricate, but particularly important, in allopolyploids. In these species, each chromosome may recombine with the slightly divergent chromosomes inherited from the other parental species (i.e., the homoeologous chromosomes) instead of or in addition to its true homologue. Whenever COs are formed between the homoeologues, they result in chromosome missegregation, aneuploidy and reduced fertility (Ramsey and Schemske 2002). Polyploidy therefore requires additional meiotic adaptations (see “Meiotic recombination between non-homologous chromosomes” section).

At least two independent pathways contribute to CO formation in plants (reviewed in Lambing et al. 2017; Mercier et al. 2015). The majority of COs in plants (Class I CO) are produced through the so-called ZMM pathway. Therefore, CO frequencies are strongly reduced in plant *zmm* mutants, such as *zip4* or *hei10* (Chelysheva et al. 2007, 2012; Shen et al. 2012; Wang et al. 2012). Class I COs are subject to interference; i.e., they are more regularly spaced along chromosomes than if they were randomly positioned. Current models suggest that this patterning could originate from the same single basic process that is responsible for the obligatory CO (Wang et al. 2015). The second class of COs (Class II COs) is a minority during WT meiosis and are not sensitive to interference (Berchowitz et al. 2007; Higgins et al. 2008; Kurzbauer et al. 2018 and ref. therein).

Running counter to these pro-CO activities, at least three independent pathways limit CO formation in plants. These rely on the activities of (1) FANCM and its cofactors (Crismani et al. 2012; Girard et al. 2014), (2) FIGL1 and its partner FLIP (Fernandes et al. 2018b; Girard et al. 2015; Hu et al. 2017; Zhang et al. 2017) and (3) RECQ4A/RECQ4B and the associated proteins TOP3α and RMI1 (Séguéla-Arnaud et al. 2015, 2016), respectively. It was recently shown that disrupting any (combination) of these pathways results in a massive increase in Class II COs in *A. thaliana* (Fernandes et al. 2018a). Current studies are investigating whether CO frequencies can be increased in crops by knocking down the anti-CO proteins (see “Increasing crossover rates in crops by knocking-out anti-crossover factors” section below).

### Patterning of meiotic COs formation

COs do not have the same probability of occurring in every spot of the genome. In most organisms, 80% of the COs are concentrated in ~ 25% of the genome (Choi et al. 2013; Saintenac et al. 2009). Domains with high CO rates (hot regions) alternate with domains where CO rates are significantly lower than the genome-wide average (cold regions). This heterogeneity in CO localization remains true regardless of the genomic scale considered (from nucleotides to chromosomal regions).

At a fine scale, plant COs usually occur close to gene promoters and terminators in regions where DNA is accessible (Choi et al. 2013; Drouaud et al. 2013; Fu et al. 2002; He et al. 2017; Wang and Copenhaver 2018). In Arabidopsis, CO hotspots are closely associated with DSB hot spots (Choi et al. 2018). In maize, however, most DSBs are formed in repetitive DNA and only the subset of DSBs that occur near genes (~ a quarter of total DSBs) are likely to contribute to CO formation (He et al. 2017); this suggests that either there is a difference in the way the genic and non-genic DSBs are formed or a difference in the way they are repaired.

A series of specific marks and sequence contexts have been shown to be associated with the formation of DSBs and/or COs (Mézard et al. 2015). For example, DSBs are preferentially formed at nucleosome-free, DNA-hypomethylated sites in both Arabidopsis and maize (Choi et al. 2018; He et al. 2017). These marks/sequences can differ between species. Thus, although DSB hotspots are associated with AT-sequence richness in Arabidopsis (Choi et al. 2018), a 20-bp-long GC-rich degenerate DNA sequence motif has recently been shown to be present in the majority of maize genic DSB hotspots (He et al. 2017). CO hotspots are also influenced by chromatin structure and can be suppressed by DNA methylation, as well as other heterochromatic modifications (Yelina et al. 2015).

At a broader chromosomal scale, in most plant species COs concentrate in distal euchromatic regions while more centrally located regions are usually poor in COs (Choulet et al. 2014; Demirci et al. 2017; Higgins et al. 2014; Lambing et al. 2017; Lukaszewski and Curtis 1993; Zahn 2018). This centromere-telomere gradient for CO frequencies can be very pronounced as in the chromosome 3B of wheat, for example. While the proximal regions show a very weak CO frequency, ~ 80% of COs are concentrated in the distal ends of the chromosome that represents only ~ 20% of chromosome length (Darrier et al. 2017; Saintenac et al. 2009). This observation is not universal, however. In Arabidopsis and rice, CO frequency is more evenly distributed (Lambing et al. 2017 and references therein). In *Allium fistulosum*, the centromere-telomere gradient is even reversed; recombination peaks in regions close to the centromere with 90% of COs occurring within the proximal 25% of homologues length engaged in recombination (Albini and Jones 1987).

In all plant species COs are suppressed at and near the functional centromeres, i.e., the sites where kinetochore attach to spindle microtubules to allow chromosome segregation (Underwood et al. 2018 and ref therein). The mechanisms for CO suppression at the centromeres and pericentromeres are poorly understood. A recent study has demonstrated that during WT meiosis in Arabidopsis, epigenetic marks, such as H3K9me2 and DNA methylation, suppress initiation of meiotic recombination in the centromeric and pericentromeric regions (Underwood et al. 2018 and ref therein). Interestingly, while methylation intensity has a major influence on CO formation in centromeric regions, the total CO number does not increase genome wide in hypomethylated mutants. For example, loss of CG DNA methylation in Arabidopsis leads to an increase in centromere-proximal COs, while pericentromeric CO are redistributed toward euchromatic distal regions (Yelina et al. 2012). In H3K9me2 and non-CG DNA mutants, CO frequency increases in pericentromeric regions and concurrently decreases in CO arms (Underwood et al. 2018). Thus, although epigenetic marks contribute to define non-recombining centromeric regions, other factors contribute to shape the global CO frequency.

In barley, Higgins et al. (2012) observed that recombination is initiated throughout the entire nucleus, albeit in a polarized manner. Recombination initiation in proximal regions occurs 2–3 h later than in the most distal regions and rarely progresses to yield COs. Because of this pronounced temporal differentiation in CO initiation, it is possible that recombination intermediates are channeled toward the CO pathways at distal sites before DSBs are formed in more interstitial regions. The timing of recombination initiation, which could be linked with the timing of DNA replication, could thus influence the CO landscape and explain why~ half of all chromosome arms do not form COs in barley (Higgins et al. 2014).

In addition to primary and secondary chromosomal structures, other factors contribute to shape the CO landscape. This is best illustrated when CO patterns between male and female meiosis are compared. CO number and distribution along chromosomes depend on sex in most but not all (e.g., tomato, barley) species (Lenormand and Dutheil 2005). The pattern can be very contrasted. In Arabidopsis, for example, CO rates in distal regions are very high in male meiosis but very low in female meiosis (Giraut et al. 2011). The mechanisms responsible for such “heterochiasmy” are not known.

### Meiotic recombination between non-homologous chromosomes

It has long been observed that CO frequencies tend to decrease between regions with increased sequence divergence (Borts and Haber 1987; Liharska et al. 1996; Ziolkowski and Henderson 2017). Local inhibition of recombination due to sequence divergence is usually attributed to the presence of mismatched base pairs within recombination intermediates. These lead to their destabilization, either directly or by triggering the MisMatch Repair (MMR) machinery. Consistent with this, a significant ~ 40% increase in CO rate was observed (for one genetic interval) in an Arabidopsis hybrid deficient for MSH2, a key MMR protein, compared to a WT hybrid (Emmanuel et al. 2006). This suggests that MSH2 acts as an anti-CO protein when homologous chromosomes are polymorphic (Emmanuel et al. 2006).

The general observation that COs tend to move away from divergent regions has been recently challenged in Arabidopsis. Ziolkowski et al. (2015) observed an increase in CO frequencies within megabase heterozygous regions juxtaposed with surrounding homozygous regions. This suggests that the mechanisms by which sequence polymorphisms affect meiotic recombination are still unclear. In addition, not all recombination intermediates have the same sensitivity to sequence divergence. In Arabidopsis, while the extra-CO in *figl1* and *recq4* is unaffected by polymorphisms in a hybrid context, this is not the case in *fancm* mutants where additional CO are only observed in pure inbred lines (see “Increasing crossover rates in crops by knocking-out anti-crossover factors” section below, however). Future studies could examine the sensitivity of the different CO pathways to polymorphisms in a set of other species and/or heterozygosity contexts to gain more understanding of the underlying mechanisms.

In allopolyploids, sequence polymorphism is not sufficient to abolish CO formation between homoeologous chromosomes. The occurrence of chiasmatic multivalents is indeed commonplace in recent and/or resynthesized allopolyploids (e.g., Ramsey and Schemske 2002; Szadkowski et al. 2010). An additional layer of control is therefore required to promote the strict formation of COs between homologous over homoeologous chromosomes (Grandont et al. 2013). The molecular mechanisms responsible for such chromosome sorting in allopolyploids remain poorly understood, except in wheat where the system (i.e., *Ph1*) is very close to being resolved (Martín et al. 2017). The *Ph1* locus, which was first described 60 years ago (Riley and Chapman 1958; Sears and Okamoto 1958), was initially associated with a cluster of cyclin-dependent-like kinases (CDKs) on chromosome 5B interrupted by a block of heterochromatin (Greer et al. 2012; Griffiths et al. 2006). Rey et al. (2017) recently revealed that the *Ph1* locus also contains an extra copy of the ZMM gene *ZIP4 (Tazip4*-*B2)* and showed that TaZIP4-B2 is responsible, at least partially, for CO suppression between homoeologous chromosomes.

Although the precise mechanism remains elusive, identification of *TaZIP4*-*B2* has opened new opportunities for plant breeding (see “The strict control of CO formation in interspecific hybrids and polyploids ensures plant fertility but limits genetic exchange between non-homologous chromosomes” section below).

## Tinkering with meiotic recombination could be crucial for speeding-up progress in breeding programs

Meiotic recombination is crucial to breeders as it ensures plant fertility and generates genetic diversity. However, the strict control mechanisms that are necessary for a proper functioning meiotic recombination can thwart breeder’s efforts to construct the desired combinations of alleles.

### Crossovers reshuffle genetic information but are limited in number

In addition to the genetic diversity resulting from the random segregation of paternal and maternal homologues during meiosis, CO formation results in new allelic combinations that may carry advantageous functional innovations. However, the low number of COs often limits the genetic variation that can be captured in plant breeding programs.

In the vast majority of species, the mean number of COs per chromosome rarely exceeds three per bivalent. This holds true irrespective of the physical size of the chromosome and despite an excess in CO precursors (Mercier et al. 2015). This limit has both direct and indirect consequences on genetic diversity because of the inherent mutagenic nature of CO formation (Rattray et al. 2015) and its influence on selection (Tiley and Burleigh 2015). The reduced nucleotide variability associated with selection is amplified in regions of low CO frequency because positive selection for a favorable allele leads to an increase in the frequency of genetically linked alleles that are dragged along in a “positive sweep.” The lower the CO frequency, the larger the region that is swept; i.e., where genetic diversity is erased. Thus, by increasing CO frequencies new favorable alleles could be introduced not only through increased allele reshuffling but also through reduced genetic variance loss in regions subjected to selection.

Only a few simulation studies have tested specifically whether higher CO frequencies would positively affect selection efficacy. McClosky and Tanksley (2013) simulated populations derived from a biparental inbred cross where the breeders selects for extreme or “transgressive” phenotypes. They observed that increased CO frequencies resulted in significant, but relatively modest gains (11%) in response to selection. Another simulation (in livestock) suggests that more substantial increases in response gains to selection would require larger increases in CO frequency (33% gain obtained with a 20-fold increase) (Battagin et al. 2016). In these two studies, however, the simulated CO rates were computed without taking in account CO interference, thereby possibly inflating the number of double (or multiple) recombinants that could be recovered in the WT populations. It thus remains to be seen whether a greater benefit of increasing CO number would be obtained by comparing a WT population in which COs are interfering with a population in which COs are no longer subject to interference (as in the anti-CO mutants described in “The molecular mechanisms of meiotic recombination” section). In addition, availability of HyperRec genotypes in a wide range of plant species (Fernandes et al. 2018a; Mieulet et al. 2018) now makes it possible to compare computer simulations with field experiments.

Finally, it must be acknowledged that increasing CO frequency/genetic diversity cannot be an end in itself. Disruption of beneficial gene combinations that already exist in elite cultivars might outweigh the advantages of increasing CO rate. Serious thought is therefore needed to determine the best way to make use of the methodologies that can be used to boost CO frequencies in plants (see “Increasing crossover rates in crops by knocking-out anti-crossover factors” section). Whereas increased COs can certainly meet a need in the context of pre-breeding, i.e., “all activities designed to identify desirable characteristics and/or genes from unadapted materials that cannot be used directly in breeding populations and to transfer these traits to an intermediate set of materials that breeders can use further in producing new varieties” (Biodiversity International and GIPB/FAO 2008), a sparing use of these approaches should, however, be considered for the subsequent steps of elite variety development.

### Beneficial combinations of alleles are preserved in CO-poor regions but deleterious mutations are difficult to get rid off

Another factor limiting the opportunities offered by meiotic recombination is the uneven distribution of meiotic COs across the genome (see “Patterning of meiotic COs formation” section). While CO-poor regions are often enriched in repeated sequences (He et al. 2017; Sato et al. 2012; Zahn 2018), this does not mean that these regions are completely deprived of genes. For example, in barley and maize, CO-poor regions contain around 20% of total gene content (Bauer et al. 2013; Mayer et al. 2012). This is even more extreme on wheat chromosome 3B where 70% of total gene content is found in CO-poor regions (Choulet et al. 2014). Some of these genes are of interest for breeders. For example, Choulet et al. (2014) showed that half of the newly identified Quantitative Traits Loci (QTLs) for yield, nitrogen use efficiency, crop height and ear emergence are located within the CO-poor regions of wheat chromosome 3B.

As CO rate is low in those regions, blocks of strong linkage disequilibrium spanning large intervals are created, with direct consequences for genetic mapping. For example, the confidence intervals of QTLs in CO-poor regions on chromosome 3B can cover hundreds of megabases (Choulet et al. 2014), which reduces the odds of identifying positional gene candidates for these QTLs. In addition, and as detailed above, selection cannot act specifically on one particular locus but affects very large numbers of neighboring loci in CO-poor regions. This process not only reduces the chance of increasing genetic diversity in these regions, it also reduces the chance of removing deleterious mutations which tend to accumulate in low recombining regions (Renaut and Rieseberg 2015; Rodgers-Melnick et al. 2015). Purging crop genomes of these deleterious mutations could be considered as a way to improve crop varieties but is currently difficult to achieve because of limited COs in these regions.

Modifying CO patterns (without increasing genome-wide CO frequency) is thus predicted to result in an increase in genetic gain (Gonen et al. 2017). In a simulation study in cattle, (Gonen et al. 2017) showed that targeting COs to non-recombining, but polymorphic regions reduced the loss of genetic variance and increased genetic gain over subsequent generations. The largest benefit was observed when the polymorphisms contributing to the traits of interest were clustered. The genetic architecture of the trait is thus essential. In this context, a local increase in CO frequency could help solve the confounding issue of linked QTLs with opposing effects, which are difficult to detect because their alleles cancel each other out (Joseph et al. 2014; Yamamoto et al. 2014). Thus, increasing CO frequency in those specific regions (rather than increasing the population size or the number of generations) could provide an interesting way to detect new loci of interest for plant breeding.

### The strict control of CO formation in interspecific hybrids and polyploids ensures plant fertility but limits genetic exchange between non-homologous chromosomes

Wild crop relatives are an important source of beneficial alleles for crop improvement (Mammadov et al. 2018 and references therein). For example, Gur and Zamir (2004) showed that introgressions of genomic regions from a wild tomato species (*Solanum pennellii*) into the genome of an elite variety increased yield 1.5-fold compared to a control. The biggest increase was obtained when the introgressions were hemizygous, which suggests the presence of linked deleterious recessive genes originating from the wild tomato. Likewise, “alien” alleles have been introgressed from > 50 species (belonging to 13 genera) into wheat (Doussinault et al. 1983; reviewed in Hajjar and Hodgkin 2007; Crespo-Herrera et al. 2017).

Despite these successful examples of introgression, mining allelic variation within genetic resource collections is limited by the mechanisms that control CO formation between homologous chromosome (see “Meiotic recombination between non-homologous chromosomes” section). For example, genome-wide COs are reduced by ~ 30% in a hybrid between tomato and *S. lycopersicoides* compared to an intraspecies hybrid (Chetelat and Meglic 2000) and down to 0–10% in regions where introgressions from related species are present in the heterozygous state (Canady et al. 2006; Demirci et al. 2017). In allopolyploids, the same genetic systems that promote the strict distribution of COs between homologues (thereby ensuring fertility and genome stability) hamper the incorporation of beneficial alleles into crop plants from their wild relatives; for example, deletion of *Ph1* or suppression of its activity was identified from the beginning as a prerequisite to transfer alien genes into wheat (e.g., Riley et al. 1968a, b; Sears 1973). Although suppression of *Ph1* activity is sufficient to promote CO formation in wheat hybrids, this does not resolve all difficulties (see “Increasing crossover rate between divergent chromosomes” section) and the number of successful introgressions (in terms of development of new elite varieties) has remained quite low (King et al. 2017).

One constraint on the use of exotic introgressions is linkage drag, i.e., fitness loss due to deleterious genes introduced along with the beneficial ones. For example, introgressions of rye into wheat frequently had negative effects on wheat baking quality (reviewed in Kumlay et al. 2003). Clearly, the larger the size of the introgressed fragments, the higher the chance of introducing undesired alleles from the non-adapted exotic germplasm that negatively alter crop performance. A second step is thus necessary to reduce the size of the introgression around the target locus. However, CO rate is usually reduced at introgression sites (Liharska et al. 1996) and this limits the chances of removing linkage drag between beneficial and undesired alleles introduced from exotic germplasms. Generally, the reduction in CO frequency is more pronounced when the introduced fragment originates from a species that is more distantly related to the recipient crop (Liharska et al. 1996). Although breeding strategies have been proposed for carrying out targeted introgressions, their implementation can prove difficult and long (see below).

## How to boost meiotic crossover formation in plants

**Fig. 1:**
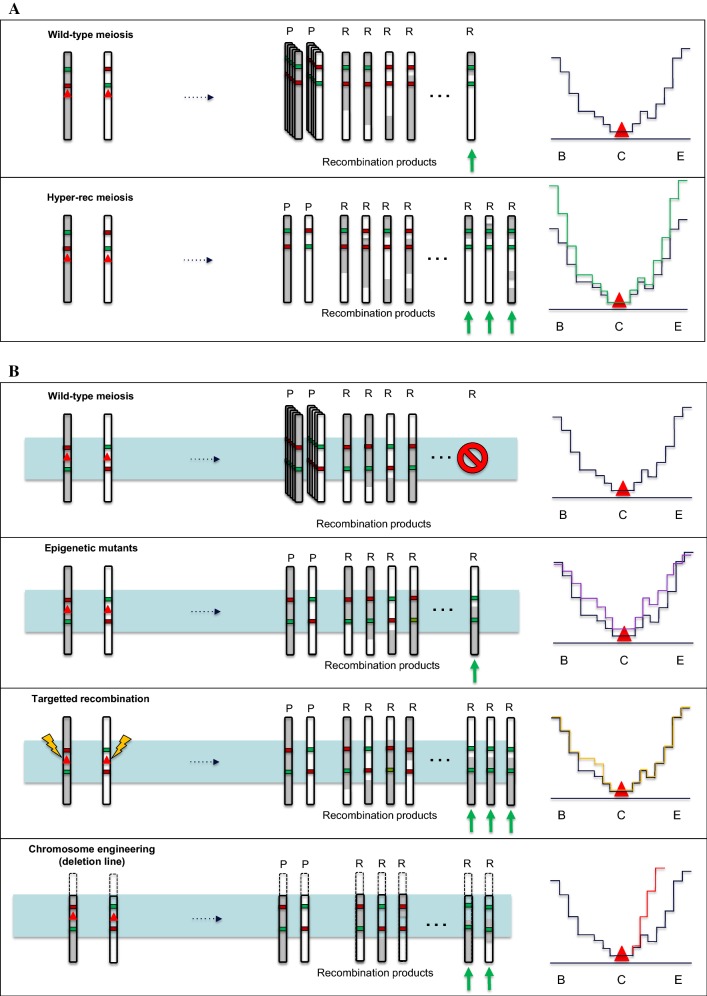

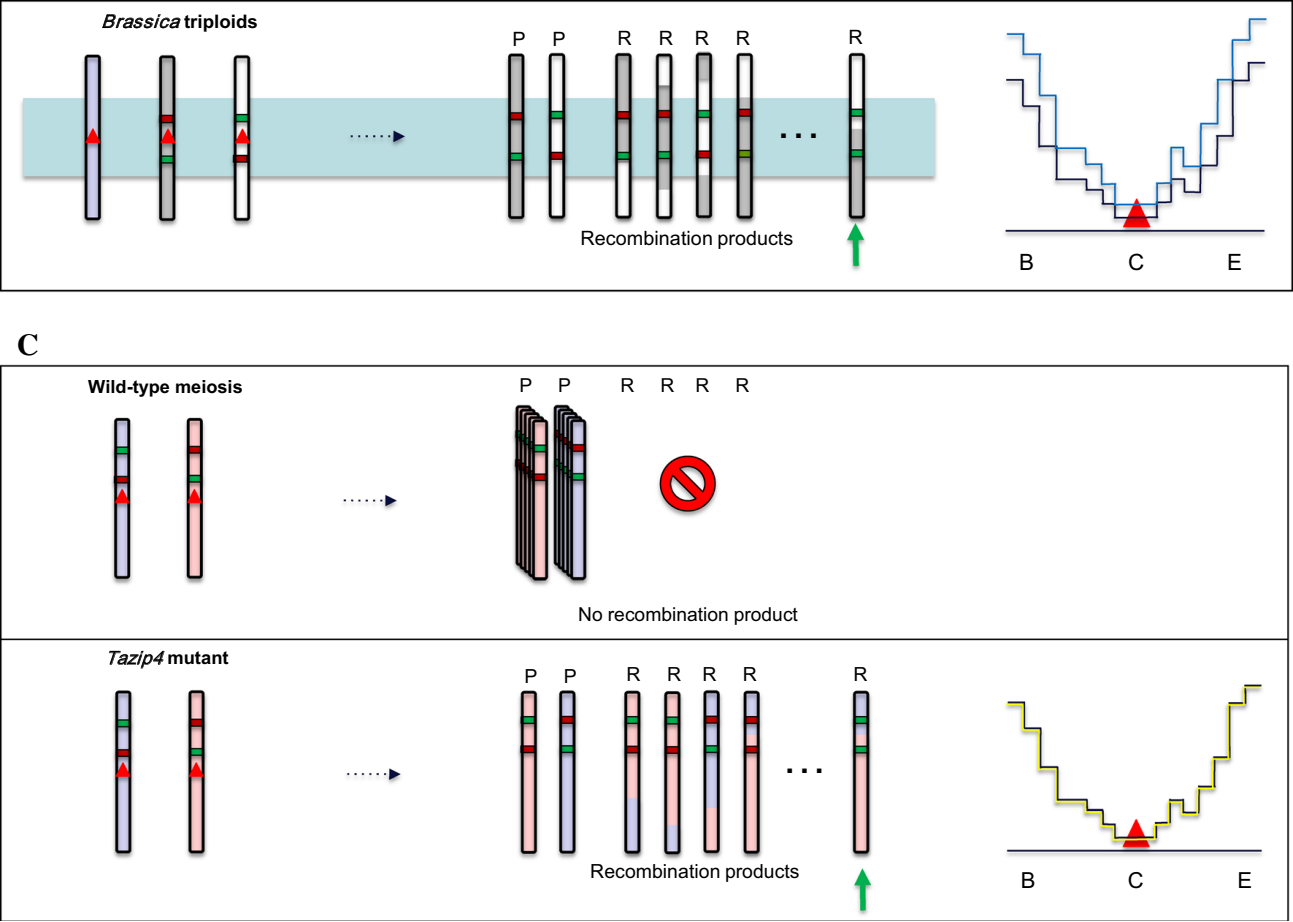
How boosting crossover formation can benefit plant breeding. This schematic illustrates hybrid plants where meiotic recombination has been altered through mutagenesis or chromosome engineering approaches. On the left of each panel, the genome reshuffling occurring in the plants during meiosis is shown. For the sake of simplicity, only one homologous chromosome pair is shown per plant (the sister chromatids are not represented). Again, for sake of simplicity only one CO occurs in WT meiosis. Examples of the resulting parental (P) or recombinant chromosomes (R) are shown. Colored segments indicate alleles that influence, positively (green) or negatively (red) a trait under selection. A green arrow points to the recombinant(s) with the desired combination of alleles. In panel B, an additional, non-paired homoeologous chromosome is represented in light blue. In panel C, pairs of homoeologous chromosomes (light blue and light red) instead of homologous chromosomes (white and gray) are represented. On the right of each panel, the CO frequency (*y* axis) along the chromosome arm (B standing for chromosome begin, C for centromere and E for chromosome end on the *x* axis) in the corresponding plant is shown. For the sake of simplicity, CO frequency in WT (black) is arbitrarily low in centromeric and pericentromeric regions (red triangle) and high in distal regions. The expected meiotic CO landscape in the various mutants or chromosome-engineered plants is represented by the different colors (color figure online)

Over the years, an increasing number of studies have addressed how fundamental knowledge on the underlying mechanisms of meiotic CO formation could be used for plant breeding purposes (Fig. 1). While recent studies confirmed that CO frequencies can be sharply increased in crops, altering meiotic CO distribution in cultivated species remains a longer-term prospect.

### Increasing crossover rates in crops by knocking-out anti-crossover factors

As described above (“The molecular mechanisms of meiotic recombination” section), a series of genetic screens identified the negative regulators of CO frequency in *A. thaliana* (Crismani et al. 2012; Fernandes et al. 2018a; Girard et al. 2014, 2015; Séguéla-Arnaud et al. 2015). Recent work has now established that their orthologues in crops play the same role.

The very first anti-CO protein to be identified through these screens was FANCM (Fanconi Anemia Complementation Group M). In Arabidopsis, mutations in *AtFANCM* lead to a ~threefold increase in COs and are sufficient to restore bivalent formation in CO-defective mutants to a level indistinguishable from WT (Crismani et al. 2012). In crops, the effect of *fancm* was first assessed in diploid and allotetraploid Brassicas (Blary et al. 2018). A TILLING approach identified nonsense and missense mutations in the single copy of *FANCM* present in diploid *B. rapa* (*BraA.FANCM*) and in the two copies of *FANCM* present in allotetraploid *B. napus (BnaA.FANCM* and *BnaC.FANCM,* respectively). In *B. rapa,* a threefold increase in COs was obtained by knocking-out *BraA.FANCM* in a plant deficient for the main (class I) CO pathway; as in Arabidopsis, the extra COs were sufficient to restore bivalent formation to a WT level in this plant (Blary et al. 2018). A less pronounced, but consistent increase in CO frequency (~ 30%) was observed in *B. napus.* This result was consistent with the fact that one of the mutations used in *B. napus* retained residual anti-CO activity. Despite this, Blary et al. (2018) repeatedly measured a significant increase in CO frequency between homologous (in allotetraploid AACC) and homoeologous (in allohaploid AC) chromosomes in the *B. napus fancm* mutant compared to WT. It is unknown, however, whether the extra COs observed in allohaploids were formed between homoeologous regions or between homologous regions duplicated on homoeologous chromosomes as a consequence of pervasive fixed homoeologous exchanges in the *B. napus* genome (Chalhoub et al. 2014; Lloyd et al. 2018a; Samans et al. 2017; Sun et al. 2017). More recently, the boosting effect of the *fancm* mutation on COs was confirmed in two other crops by Mieulet et al. (2018) who reported a ~ twofold increase in CO frequencies in rice (*Oryza sativa*) and pea (*Pisum sativum*) hybrids deficient for FANCM compared to WT hybrids. Interestingly, these results contrast with those obtained in Arabidopsis where *fancm* mutations had no effect in hybrids (see “Meiotic recombination between non-homologous chromosomes” section above). As pointed out by Mieulet et al. (2018), this apparent discrepancy may simply reflect a difference in SNP density, which is much lower in rice and pea than in Arabidopsis.

In Arabidopsis, RECQ4 is the strongest anti-CO factor identified so far with RECQ4 knockouts (KOs) resulting in a ~ fourfold increase in CO frequency genome wide. Mieulet et al. (2018) have just confirmed that the *recq4* mutation increases COs ~ threefold in rice, pea and tomato (*Solanum lycopersium*). Altogether, these results suggest that manipulating RECQ4 may be a versatile tool for boosting CO frequency in crops. However, and in contrast to FANCM, the genes encoding RECQ4 tend to be present in multiple copies that have been retained from past polyploidy events (true in sunflower, lettuce, poplar, Brassica crops) which may make the transfer more complicated. In Arabidopsis, the highest increase in CO frequency (7.8-fold increase) was obtained by combining the *recq4* mutations with a mutation in the *FIGL1* gene (Fernandes et al. 2018a). However, this approach is unlikely to produce positive results in crops as the *figl1* mutation was recently shown to induce sterility in rice, pea and tomato (Mieulet et al. 2018; Zhang et al. 2017) in contrast to Arabidopsis (Girard et al. 2015).

Another synergistic effect was obtained in Arabidopsis by combining the *recq4* mutations with an extra copy of *HEI10*, one of the ZMM proteins (Serra et al. 2017). Interestingly, the extra COs observed by increasing HEI10 dosage originate from the class I pathway (Ziolkowski et al. 2017), while those produced by the *recq4* mutations are class II COs (Séguéla-Arnaud et al. 2015). Future studies could investigate whether over-expression of HEI10, alone or in combination with *recq4* mutations, could increase CO frequency in crops.

This recent progress in translational biology is an important step forward toward the use of hyper-recombinants mutants in plant breeding. However, a number of issues must still be considered.

A first question concerns the practicality and ease of implementing the technologies that can be used to produce hyper-recombinant plants. For example, TILLING, which relies on chemical mutagenesis followed by high-throughput screening for point mutations, is applicable to all plant species that can be mutagenized and is exempt from the biosafety regulations imposed on genetically modified organisms. The ease with which nonsense mutations can be identified in target genes has been improved thanks to next generation sequencing (NGS) technologies combined with pooling strategies (Gilchrist et al. 2013; Tsai et al. 2011) and/or the development of sequenced mutant populations in crops (Krasileva et al. 2017). Two of the main drawbacks of TILLING are that: (1) the isolation of multiple mutants requires laborious and time-consuming crosses (e.g., Blary et al. 2018) and (2) chemical and/or physical mutagenesis generates a large number of off-target mutations that are not desirable from a plant breeding perspective. More targeted approaches, such as gene editing through CRISPR-Cas9, have the potential to avoid off-target mutations while co-targeting several genes simultaneously, in particular the homoeologous copies of a gene in an allopolyploid (Braatz et al. 2017; Brooks et al. 2014; Li et al. 2016; Wang et al. 2014; Yang et al. 2017). However, CRISPR-Cas9 relies on transgenesis, which is not always efficient, or even feasible, for many plant species. In addition, legal uncertainty (recently illustrated by the decision to subject CRISPR-Cas9 to the same stringent regulations as conventional genetically modified organisms in Europe) and the risk of non-acceptance of these technologies by consumers restrict the applicability of these new plant breeding technologies (Ishii and Araki 2016).

A second issue relates to the genetic characteristics of the induced KOs. The recessive nature of the mutations induced through TILLING and CRISPR-Cas9 approaches is somewhat limiting as complex crossing schemes and genotyping steps are necessary to produce homozygous mutants. This is slightly less of a concern with CRISPR-CAS9 as plants harboring edited (mutant) alleles at all target genes can potentially be recovered from the very first generations (e.g., Braatz et al. 2017). Gene expression suppression through virus-induced gene silencing (VIGS) offers the possibility of knocking-down meiotic recombination genes in a dominant way by taking advantage of plant defense mechanisms against viral infection. However, reduced expression of the target genes through VIGS is not necessarily complete. As illustrated in Blary et al. (2018), residual anti-CO activity (for FANCM) was sufficient to limit the magnitude of the CO increase in the hyper-recombinant genotype, thus raising doubts about the usefulness of hypomorphic phenotypes, except when KO mutations induce lethality or sterility. The generation of an allelic series of hypomorphic mutants with TILLING or incomplete abolition of gene expression via VIGS may thus be one avenue to explore for using *figl1* mutants in crops.

The potential to boost CO frequencies in a tissue-specific and reversible way will also be key for acceptability. Although backcrosses can be used to get rid of mutagenized (TILLING) or edited (CRISPR) target genes, breeders could be reticent to KO anti-CO factors as these genes also play somatic roles, notably in genome stability (Knoll and Puchta 2011). Although the use of VIGS with tissue-specific or inducible promoters (for example, a meiosis-specific promoter such as DMC1 (Klimyuk and Jones 1997) could be an option for transiently reducing the expression of the target gene and its homoeologues in reproductive tissue, it remains to be seen whether producing hyper-recombinants is feasible through this approach.

### Altering crossover distribution

Although increasing CO frequencies in proximal low recombining regions is of upmost importance from a plant breeding perspective (see “Beneficial combinations of alleles are preserved in CO poor regions but deleterious mutations are difficult to get rid-off” section), whether this can be achieved using anti-CO KOs may not be possible. In Arabidopsis and rice, for example, the extra COs produced in all anti-CO mutants only occurred in regions where WT COs already happen (Fernandes et al. 2018a; Mieulet et al. 2018) (Fig. 1). The same pattern was observed in Arabidopsis plants in which *recq4* mutations were combined with an extra copy of HEI10 (Serra et al. 2017) Whether the same holds true in crop genomes where large interstitial regions are depleted but not completely lacking COs remains to be established. Thus, it would make sense to look for genes or methods that could be used to specifically increase CO numbers in proximal regions.

A first approach would be to tinker with the epigenetic marks that have been shown to influence CO distribution (see “Patterning of meiotic COs formation” section). Indeed, altered methylation patterns and heterochromatin marks show interesting potential for unlocking pericentromeric CO formation. However, reports of pleiotropic developmental defects in hypomethylation mutants of rice and tomato (Corem et al. 2018; Hu et al. 2014) suggest that caution may be needed when crop improvement is the primary objective. In addition, the extent to which the results obtained in Arabidopsis will transfer to crops is not known. The inability to recover lines that are both fertile and strongly hypomethylated in crops (for example, in maize see Li et al. 2014) suggests that the interaction between epigenetic marks and CO frequency will also be more difficult to assess.

A second approach is to fine-tune the CO number very locally, according to a specific need, through direct targeting of CO formation. In *S. cerevisiae,* several approaches were tested to induce DSB formation at specific sites on the genome using SPO11 fusions with a variety of different DNA-binding factors: full-length DNA-binding proteins, zinc fingers (ZFs), transcription activator-like effector (TALE) modules, nuclease-dead Cas9 (Pecina et al. 2002; Sarno et al. 2017). Although an increase in CO increase (2.3- to 6.3-fold increase at the targeted loci, depending on the constructs and target sites) was repeatedly observed, the centromeric regions remained refractory to the targeted CO increase (Sarno et al. 2017). The approach remains promising, however, and may be worth testing in model plants and crops with large proximal CO-poor regions. Indeed, the lack of DSBs is not necessarily limiting for CO formation during plant meiosis (He et al. 2017; Higgins et al. 2012); in contrast to *S. cerevisiae*, only a small fraction of the extra induced DSBs would be repaired as COs.

### Increasing crossover rate between divergent chromosomes

Tinkering with the mechanisms that control homoeologous CO frequency has been a reality since the late 1960s, at least in wheat (Riley et al. 1968a, b; Sears 1973). Suppression of *Ph1* activity has been instrumental in developing approaches based on precise CO formation to exploit the untapped genetic variation present in relatives of wheat and develop new varieties.

Historically, suppression of *Ph1* activity was achieved using a 70 Mb large deletion on the long arm of chromosome 5B, i.e., the *ph1b* mutation. Although extensively used for introgression purpose (Riley et al. 1968a; Sears 1977; Zhao et al. 2013), *ph1b* mutants tend to accumulate extensive chromosome rearrangements due to meiotic exchanges between wheat A, B and D homoeologous chromosomes (Sánchez-Morán et al. 2001). As these exchanges lead to infertility, only a few new wheat varieties have been developed from introgression lines.

Detailed molecular characterization of the *Ph1* locus has opened new avenues for improving alien introgression into wheat. First, identification of CDKs within *Ph1* led (Knight et al. 2010) to tests of the potential of okadaic acid, a potent protein serine/threonine phosphatase inhibitor, to phenocopy the *ph1b* mutation in wheat interspecific hybrids. The authors observed that okadaic acid applied on detached tillers led to CO formation in a wheat–rye interspecific hybrid despite the presence of *Ph1* (Knight et al. 2010). This is, to the best of our knowledge, one of the first examples of the use of a chemical agent as a vector for increasing CO rates. Similar chemical-based approaches could provide a way to reversibly allow homoeologous CO formation and thus maintain fertility and genome stability once the desired recombinants are obtained. Implementing this type of strategy on a larger scale, however, will require the development of a practical way to apply the treatment.

More recently, mutagenesis approaches targeting the extra copy of *ZIP4* (*Tazip4*-*B2*) located within the *Ph1* locus have been performed to identify alternative mutants that phenocopy *ph1b* mutations with less adverse effects on genome stability. Rey et al. (2017, 2018) successfully identified *Tazip4*-*B2* EMS (through TILLING) and *Tazip4*-*B2* CRISPR mutants showed similar CO levels in hybrids with *Aegilops variabilis* as previously reported in *ph1b* wheat–*Ae. variabilis* hybrids. The *Tazip4*-*B2* EMS mutants in wheat did not show multivalents at metaphase I in contrast to *Ph1* deletion mutants (Roberts et al. 1999). Use of *Tazip4*-*B2* EMS mutants rather than *Ph1* deletion mutants is therefore more suited for introgression in wheat hybrids.

Some alternatives to mutagenesis have been proposed for overriding *Ph1* activity. These are based on the use of genes found in wheat wild relatives (Sears 1976) such as *Amblyopyrum muticum* (Dover and Riley 1972; see also King et al. 2017 and ref therein) and *Aegilops speltoides* (Feldman and Mello-Sampayo 1967; Riley et al. 1961; Li et al. 2017 and ref therein), which suppress *Ph1*. The promotion of COs between homoeologues can be obtained either directly in F1 hybrids (King et al. 2017) or after the suppressor of *Ph1* has been introgressed into wheat. The first approach is straightforward, but limited to the genotypes that are able to override *Ph1* activity. The second approach is more versatile, but longer and more difficult to implement. Recently, Li et al. (2017) used marker-assisted selection to introgress a dominant suppressor of *Ph1* (*Su1*-*Ph1*) from *Aegilops speltoides* into hexaploid and tetraploid wheat. Although the presence of the introgression suppressed *Ph1* activity in the tetraploid background, this was not the case in hexaploid wheat. The authors suggested that a complementary gene, absent in the hexaploid wheat background and in the introgressed fragment, is necessary for *Su1*-*Ph1* suppression activity. Although the use of a natural allele rather than mutagenesis could simplify the introgression process, *Su1*-*Ph1* is less effective than *ph1b* mutation at inducing homoeologous COs.

Finally, translational biology approaches have also been carried out to counteract the effects of sequence divergence on meiotic COs in wild tomato hybrids. Considering the role of the DNA mismatch repair (MMR) system (see “Meiotic recombination between non-homologous chromosomes” section), (Tam et al. 2011) assessed how frequently a chromosome introgressed from a wild tomato (*Solanum lycopersicoides*) into a cultivated tomato (*Solanum lycopersicum*) forms CO with its orthologue counterparts in *mmr* mutant backgrounds. In order to do so, the authors used transgenic lines with RNAi gene silencing and/or dominant negative constructs to target two key MMR genes (*MSH2* and *MSH7*). A modest (average rate of 17.8%) but significant increase in CO frequency between related but slightly divergent chromosomes was repeatedly observed in the obtained mutants, although with considerable variation between the transgenic population and the marker interval considered.

As stated above (“The strict control of CO formation in interspecific hybrids and polyploids ensures plant fertility but limits genetic exchange between non-homologous chromosomes”), primary recombinant chromosomes resulting from COs between the exotic and recipient chromosomes must usually be reshaped to remove linkage drag. From the very beginning, Sears (1981) proposed a two-step approach to reduce the size of introgressions. As this approach has been recently reviewed in Lukaszewski (2016), we will only briefly describe it here. In this strategy pairs of recombinant chromosomes, each carrying the desired introgression but in a different configuration are selected: one primary recombinant consisting in a wheat proximal chromosome and a distal alien segment and a second primary recombinant showing the inverse configuration—i.e., proximal alien chromosome with a distal wheat segment. Once brought together (in the presence of *Ph1*), these two chromosomes will form CO(s) mainly in the only region that is shared between them, i.e., the segment in-between the two primary breakpoints, which contain the gene to be introgressed, resulting in intercalary alien introgressions. This approach has been successfully applied to eliminate a quality defect during bread making associated with an introgression from rye (Lukaszewski 2000). Alternatively, the chromosome carrying the desired introgression could be subjected to two (or more) cycles of undirected homoeologous CO formation induced by the absence of *Phl* to generate CO in the introgressed fragment (Luo et al. 1996).

The success of such an approach thus depends on (1) the position of the breakpoints in the recombinant chromosomes, (2) the position of the segment containing the gene of interest. Depending on its size and location, to precisely introgress a chromosomal fragment, a large number of primary recombinants must be isolated to increase the chance of recovering the desired CO breakpoints (Lukaszewski 2016). A large screening population and an efficient selection methodology are needed to recover these rare primary recombinants.

Advances in sequencing and genotyping technologies now extend the opportunities for detecting and isolating “tailored” introgressions between crops and their wild relatives, thus opening the way to the massive generation of introgression materials for future (foreseen and unforeseen) needs, i.e., “Introgressiomics” (Prohens et al. 2017). For example, combined use of the *Ph1* suppressor action of *Amblyopyrum muticum* genes with genotyping arrays allowed hundreds of introgressions of *Amblyopyrum muticum* into wheat to be detected and characterized at the whole-genome level (King et al. 2017). Increasing the efficiency of introgression approaches will be necessary to close the gap between the rapid unveiling of new and favorable genetic variations in gene bank accessions (Sehgal et al. 2015) and the concrete application of this variation in plant breeding programs. Interestingly, speed breeding seems to be compatible with the use of *ph1b*. Increasing the number of generations and thus the number of CO constitutes a straightforward option to increase the chances of recovering the desired recombinants (Watson et al. 2018).

### Chromosome engineering to alter the centromere-telomere gradient of crossover frequencies

Given that primary chromosomal structure is a strong determinant for CO localization (see “Patterning of meiotic COs formation” section), multiple studies explored the possibility of modifying chromosomal primary structure to alter the centromere-telomere gradient for CO frequencies.

In wheat (Lukaszewski et al. 2012) and a wheat–rye hybrid (Lukaszewski 2008), inversion of a chromosome arm resulted in the inversion of the CO distribution pattern. As a result, originally distal and CO-prone regions were moved close to the centromere. Although the findings of this study were very important for improving our basic knowledge, it was of limited assistance in plant breeding because COs continued to occur in the same regions.

In contrast, placing proximal low recombining regions closer to the chromosome end results in higher CO frequencies in these regions. This can be achieved by deleting the most distal part of a chromosome as demonstrated by Jones et al. (2002) in wheat. As a result, CO frequency significantly increased in this newly defined terminal segment compared to the CO rate in the same segment of the complete arm. Importantly, heterozygosity for the distal deletion prevents CO formation in the remaining arm. Thus, irrespective of the size of the deletion, COs exclusively form between equal sized chromosomes (Lukaszewski 1997), which hinders the possible applications of this approach.

Using a different strategy, Ederveen et al. (2015) irradiated pollen in Arabidopsis to generate large structural variations (deletion and inversion) where CO formation cannot occur. In most cases, CO homoeostasis resulted in an increase in CO frequencies in regions proximal to the structural variant. Although the largest increase was observed in regions close to the telomere, Ederveen et al. (2015) nonetheless noted a maximum increase of just over 150% in CO frequencies in intervals proximal to the centromere.

Although quite effective for increasing CO frequency in designated chromosomal segments, practical use of these lines needs to be well thought through. In deletion lines for example, there was an increase in genome reshuffling in proximal regions at the expense of allele diversity in the distal deleted arm (Fig. 1).

### Use of polyploidy to alter crossover patterns

While the development and use of specific plant cytogenetic resources such as deletion or inversion lines can be quite demanding (and not feasible in all species), in some crops manipulation of the ploidy level may be a straightforward strategy for altering CO formation.

The link between ploidy level and CO frequencies has been explicitly studied in *Brassica* interspecific hybrids, which provide opportunities to combine different genomes at different ploidy levels. In Leflon et al. (2010), the authors observed an unexpected boost in CO frequencies between pairs of homologous A chromosomes in allotriploid AAC hybrids. This CO increase occurs genome wide and is more pronounced in female compared to male meiosis (3.4- vs. 1.8-fold) (Leflon et al. 2010; Nicolas et al. 2009; Pelé et al. 2017). In male meiosis, the increase is partly driven by the first CO pathway (Pelé et al. 2017). Indeed, CO number was not only increased in the AAC triploids but the CO landscape was also dramatically reshaped, notably around centromeric regions which experienced a boost in CO frequency (Pelé et al. 2017).

Interestingly, the number and nature of the chromosomes which remain univalent were shown to modulate CO frequencies; Suay et al. (2014) observed that the addition of one specific chromosome is sufficient to boost CO frequencies while addition of three other chromosomes had no effect. Very recently, Harper et al. (2018) demonstrated that the presence of supernumerary chromosomes in *Lolium perenne* also results in an increase in CO frequency in the low recombining fraction of the genome. Pecinka et al. 2011 also observed that CO frequencies increased in newly formed polyploids, including autotetraploids (*A.thaliana* x *A.thaliana*) and allotetraploids (*A.thaliana* × *A. arenosa*), compared to diploid *A. thaliana*, with all the plants sharing an identical genetic background. Altogether, these results suggest that the underlying mechanisms originate from basic processes that are not lineage-specific but broadly shared among (plant) species. The exact nature of these mechanisms remains to be determined.

In the meantime, *Brassica* AAC or ACC triploids can already be used to reintroduce diversity from *B. rapa* and *B. oleracea* into modern *B. napus* cultivars that have undergone a notable decline in genetic diversity due to intense selection (Hasan et al. 2006; Qian et al. 2014). Interspecific hybridization has been widely used to develop Chinese *B. napus* adapted to local conditions using introgressions from Asian *B. rapa* (Qian et al. 2006). Tickling with ploidy level or additional set of chromosomes may open similar opportunities for breeders, even outside of the *Brassicas*.

### Using the environment to influence crossover patterning

An alternative to the use of mutants or specific plant cytogenetic resources, modification of environmental conditions can provide, to some extent, a simple and cheap way to tinker with meiotic recombination. Although little is known about the underlying mechanisms, it has long been observed that meiotic recombination shows plasticity in response to external factors such as temperature and nutrient composition.

The effect of temperature on CO formation has been studied in numerous species. Various trends have been reported (reviewed in Bomblies et al. 2015), in particular U-shaped response curves to temperature where CO frequency is minimum at mid-range temperatures and increases when temperature increases or decreases within a biologically relevant range. In Arabidopsis, CO frequencies were, for example, ~ 10% higher at the extreme of the fertile temperature range (8–28 °C) (Lloyd et al. 2018b). Only the class I pathway contributed to the extra COs. It was shown that this increase in CO frequencies at 28 °C was not due to an increase in CO precursors (the DSBs) and did not reflect a generalized response to stress because NaCl treatment did not induce an increase in CO frequency (Modliszewski et al. 2018).

In barley, CO frequencies and also CO localization are modified due to temperature changes. Interestingly, in contrast to Arabidopsis, the extra COs observed in barley originate from the class II pathway. For class I COs only the position but not the frequency is modified. Elevating the growth temperature by 10 °C in male meiosis resulted in a shift in relative distribution of class I COs toward the centromeric regions. However, the centromere itself and the regions around it were not sensitive to the effect of temperature (Phillips et al. 2015).

Although temperature change results in relatively modest CO pattern variation, it could nonetheless be a cost-effective way to modify CO rates and or distribution, provided that fertility is not negatively impacted. A better understanding of the association between temperature and CO frequencies in other (crop) species, especially polyploids, would thus not only be relevant from a mechanistic and evolutionary perspective, but could also be beneficial to plant breeding.

Along with temperature, other factors also affect CO rates. For example, the impact of nutrition, notably potassium or phosphate content, on homologous and homoeologous CO frequency was highlighted in several studies (Grant 1952; Law 1963). It is difficult, however, to disentangle the effect of specific elements on meiotic recombination from a more generalized response caused by the nutritional status on the plant. Nevertheless, even if the mechanisms are not well understood, knowledge from classic studies can be of great use for plant breeding. For example, it was recently shown that magnesium could be provided to *ph1* mutants in wheat–rye or wheat–*Ae. variabilis* hybrids to further increase homoeologous CO frequency (Martín et al. 2017; Rey et al. 2018). This could be particularly helpful for targeted introgression strategies where formation of homoeologous COs is very limiting (see above).

## Conclusion

The adoption of new phenotypic, genotypic and cytogenetic technologies in plant breeding has been key for accelerating the development of new varieties. Technologies aimed at controlling CO formation have the potential to harness the portion of genetic diversity that remains inaccessible to breeders. Thus, these strategies could contribute to accelerate genetic gains; however, practical applications of these technologies are few. This is partly due to the challenge of translating the knowledge gained in model species to crops, especially those that are polyploid. The emergence of efficient genome editing technologies will undoubtedly speed up the process. A next necessary step will be to assess, though concrete case studies and simulations, at which stage in a breeding program tinkering with CO formation can maximize genetic gain and how well this approach integrates with other breeding tools.

### Author contribution statement

AB drafted the manuscript and EJ revised the manuscript. All authors listed have made a substantial, direct and intellectual contribution to the work and approved it for publication.
